# Mast cells: from lipid droplets to lipid mediators

**DOI:** 10.1042/CS20120602

**Published:** 2013-04-12

**Authors:** Andrea Dichlberger, Petri T. Kovanen, Wolfgang J. Schneider

**Affiliations:** *Wihuri Research Institute, Helsinki, Finland, Kalliolinnantie 4, 00140 Helsinki, Finland; †Department of Medical Biochemistry, Medical University Vienna, Max F. Perutz Laboratories, Dr. Bohrgasse 9/2, 1030 Vienna, Austria

**Keywords:** arachidonic acid, eicosanoid, lipid droplet, lipid mediator, mast cell, triacylglycerol (triglyceride), AA, arachidonic acid, ACSL, acyl-CoA synthetase, ATGL, adipose triglyceride lipase, CE, cholesteryl esters, COX, cyclo-oxygenase, DAG, diacylglycerol, DGAT2, DAG-acyltransferase 2, EET, epoxyeicosatrienoic acid, ER, endoplasmic reticulum, HETE, hydroxyeicosatetraenoic acid, HPETE, hydroperoxyeicosatetraenoic acid, HSL, hormone-sensitive lipase, IL, interleukin, KITLG, KIT ligand, LD, lipid droplet, LDL, low-density lipoprotein, LO, lipoxygenase, LT, leukotriene, MC, mast cell, BMMC, bone-marrow-derived MC, MAG, monoacylglycerol, MGL, monoglyceride lipase, PAF, platelet-activating factor, PC, phosphatidylcholine, PE, phosphatidylethanolamine, PG, prostaglandin, PGHS, PGH synthase, PI, phosphatidylinositol, PL, phospholipid, PLA_2_, phospholipase A_2_, cPLA_2_, cytosolic PLA_2_, sPLA_2_, secretory PLA_2_, TAG, triacylglyercol, VLDL, very-low-density lipoprotein

## Abstract

LDs (lipid droplets) are metabolically highly active intracellular organelles. The lipid and protein profiles of LDs are cell-type-specific, and they undergo dynamic variation upon changes in the physiological state of a cell. It is well known that the main function of the LDs in adipocytes is to ensure energy supply and to maintain lipid homoeostasis in the body. In contrast, LDs in inflammatory cells have been implicated in eicosanoid biosynthesis, particularly under inflammatory conditions, thereby enabling them to regulate immune responses. Human mast cells are potent effector cells of the innate immune system, and the triacylglycerol (triglyceride) stores of their cytoplasmic LDs have been shown to contain large amounts of arachidonic acid, the main precursor of pro-inflammatory eicosanoids. In the present review, we discuss the current knowledge about the formation and function of LDs in inflammatory cells with specific emphasis on arachidonic acid and eicosanoid metabolism. On the basis of findings reported previously and our new observations, we propose a model in which lipolysis of LD-triacylglycerols provides arachidonic acid for lipid mediator generation in human mast cells.

## GENERAL FEATURES OF LIPID DROPLET BIOLOGY

LDs (lipid droplets) are dynamic organelles in the cytoplasm of many cell types. They are most easily recognized and visible in adipocytes and in macrophage foam cells, two types of cell in which the biology of LDs has been studied most extensively. In these and other cells, such as neutrophils and eosinophils, LDs have been shown to contain a large range of lipids and to be associated with an ever-growing number of newly discovered regulatory proteins and enzymes (reviewed in, for example, [1–7]). Originally, LDs were modelled as simple structures analogous to lipoprotein particles, i.e. consisting of a core of lipid esters and a surface lipid monolayer harbouring resident and exchangeable specific proteins. In adipocytes, the main component of the neutral lipid core of LDs are TAGs [triacylglyercols (triglycerides)], whereas, in macrophages/foam cells, the core consists mainly of CEs (cholesteryl esters). In addition, DAGs (diacylglycerols), retinylesters [7–9] and ether lipids have been reported to be components of the LD cores in different cell types [10,11]. The LD surface monolayer is viewed as consisting of PLs (phospholipids), free cholesterol and lysophospholipids. However, it is likely that many minor lipid components of both the core and the surface layer of LDs have not yet been identified. This assumption appears to be supported by a growing body of experimental evidence, suggesting that the surface monolayer model of LDs is too simple. Further evidence for this notion is provided by recent biophysical studies of circulating lipoproteins, notably of LDL (low-density lipoprotein) particles [12] and/or VLDL (very-low-density lipoprotein) [13]. These investigations show that the resident apolipoprotein of these particles, ApoB (apolipoprotein B)-100, probably interacts in a dynamic fashion not only with the molecules of the surface PL layer, but also with components of the neutral lipid core. This property facilitates the formation of an interfacial third layer between the two lipid compartments [12] and, owing to the inherent conformational flexibility of the protein, permits dynamic adaptation to the different sizes of VLDL and LDL required for stabilization of the particles [13]. Interestingly, comparative proteomic studies of LDs in a variety of species, e.g. in yeast, *Drosophila* and several mammalian species (for example [4]), suggest that the core of LDs consists of several concentric layers of steryl esters which surround an inner core of TAG molecules. Indeed, evidence for such a model has been provided for the yeast *Saccharomyces cerevisiae* [14]. Other models perceive LDs as being built of curled ER (endoplasmic reticulum) with associated ribosomes immersed in secreted TAGs/CEs, covered by a PL monolayer that, in fact, may be the cytoplasmic leaflet of the ER [15,16]. This multitude of suggested models probably reflects the observed large degree of inhomogeneities in size and contents of LDs, their wide variety of functions and their adaptation to different and/or rapidly changing metabolic conditions occurring in the LD-containing cell types. To avoid confusion, it is important to note that, depending on the emphasis of the respective studies, similar or identical structures have been referred to as fat bodies, spherosomes or lipid bodies in the literature. In addition, LDs of adipocytes have been termed adiposomes [17–19], which unfortunately is also the name chosen in some reports for adipocyte-secreted microvesicles [20,21]. In the present review, we designate the lipid-filled cytoplasmic structures and/or organelles as LDs.

Our knowledge about the lipid components of LDs, and also that about the LD proteome facilitating and/or regulating the assembly and degradation of LDs as well as about the utilization of specific molecules of LDs, is far from complete. The metabolic functions of LDs can be grouped into anabolic reactions, which include fatty acid synthesis and activation, and biosynthesis of sterols and TAG molecules, and catabolic reactions, which include hydrolysis, mobilization and further metabolism of the various components, particularly the fatty acids of PLs, steryl esters and TAGs. The proteins identified early on as actors in this dynamic picture include the PAT proteins perilipin/adipophilin/TIP-47, S3-12 and OXPAT (for a suggested nomenclature of LD proteins, see [22]), certain Rab proteins [23,24] and caveolins [24–27], a large number of lipid metabolic enzymes, lipolysis-modulating proteins and lipid transport proteins, and kinases and phosphatases acting on lipids and proteins (reviewed, for example, in [1,6]). Moreover, it is intriguing that an increasing number of proteins classically defined as residents of other cellular membranes and/or organelles have been proposed or demonstrated to be components of LDs. Since the ‘foreign’ proteins or subsets thereof are consistently co-isolated with highly purified LDs, it has been argued that LDs have tight associations and interactions with mitochondria, ER domains, endosomes and peroxisomes [3,16,28,29]. Such associations would enable LDs to perform a multitude of functions traditionally assigned to other subcellular organelles. The metabolic features of LDs as sites of storage and mobilization of lipids require the ability to rapidly change their complement of proteins as well as lipids in response to extracellular cues. Indeed, many of the above-described LD components can undergo large alterations in their numbers and metabolic state, for example in their phosphorylation status [3,7], without compromising the nature of these essential organelles.

The most easily observed function of LDs, i.e. accumulation of lipids for storage via influx, has been the starting point for attempts to delineate an integrated view of LDs as important organelles for assisting intracellular protein and lipid transport, which includes the mobilization and regulated efflux of lipids [5,6,30]. The rapid turnover of key LD components can perfectly fulfil the requirements for fatty acids to fuel cells, liberate lipid components for membrane synthesis, deliver precursors for hormone synthesis and generate secretory signalling molecules (lipid mediators) via enzymatic conversion of stored precursors. Inflammatory cells, including one particularly interesting cell type, the MC (mast cell), produce and secrete powerful lipid mediators [31]. The mechanism underlying the generation of these mediators by MCs and the involvement of LDs in the process are the focus of the present review and are discussed below.

## LD FORMATION IN INFLAMMATORY CELLS

The molecular details of the processes of initiation and growth of LDs in any type of cell are still poorly understood. One reason for this shortcoming is that the experimental means to determine the mechanism and dynamics of LD formation are severely limited, for example by the fact that the size of a nascent LD is below the limits of resolution of light microscopy. Electron microscopic methodology, on the other hand, is limited to analysis of static images. Thus unambiguous experimental evidence for the mode of LD initiation is lacking. Notwithstanding this limitation, different models have been proposed, four of which can be deduced from the literature, as summarized previously in [6]. In short, these models include: (i) the ‘oiling out’ of TAGs synthesized *in situ* between the two ER leaflets [5]; (ii) bicelle formation, in which a lipid lens is excised from the ER [32]; (iii) budding of a bilayer vesicle, whose intramembranous space becomes filled with neutral lipids [6]; and (iv) LD growth by transfer of ER-derived neutral lipids into a concave depression (‘egg cup’) of the ER membrane [33]. The ‘egg cup’ model is based on data obtained by freeze–fracture electron microscopy, and in fact disputes that LDs form within the ER membrane bilayer [15]. In all of these models, LDs form towards the cytoplasmic aspect of the ER; however, LDs could also be of luminal origin [34]. Regardless of which model(s), if any, will ultimately prevail, all of these proposed mechanisms specify the ER as the key organelle for LD formation, and thus this feature appears to be the only well-established fact. In support of this conclusion, enzymes for the synthesis of a vast collection of lipids are resident proteins of the ER. Finally, a variation of the vesicle budding model posits that the droplets may never completely leave, but rather remain connected to the outer leaflet of the ER membrane [3,34], a notion which is in agreement with the cited proposals above for the internal structure of LDs [14–16].

Besides adipocytes and macrophages, the biology of the formation and functions of LDs has been studied in eosinophils and neutrophils in considerable detail. Melo et al. [35] have comprehensively summarized available data on the effects of exogenously added molecules as inducers of LD formation (see Table 1 in [35]) in various cell types. The lipid components reported to increase and/or stimulate LD formation were unsaturated, but not saturated, fatty acids, PAF (platelet-activating factor) and the eicosanoid PG (prostaglandin) D_2_ (in eosinophils only). Among the unsaturated fatty acids, early investigations dealt with the incorporation of AA (arachidonic acid), the precursor of eicosanoids, as a tracer that indicated LD growth in human lung MCs and macrophages [36,37], in neutrophilic leucocytes [38] and in eosinophils [38,39]. Accordingly, studies on the cellular uptake of radiolabelled AA, as analysed by electron microscopic autoradiography, showed that exogenous AA was predominantly incorporated into LDs of MCs, macrophages, neutrophils, eosinophils and epithelial cells [36,38,40]. Lipid analysis of purified LDs provided confirmatory evidence that these organelles are reservoirs of arachidonoyl lipids. Importantly, eosinophils incorporated AA mainly into the PL pool [38], whereas in monocytes/macrophages neutral lipids appeared to be the major target of exogenous AA [41,42]. Comparative analysis of different human circulating inflammatory cells (monocytes, neutrophils, eosinophils and platelets) and tissue cells (lung macrophages and MCs) revealed macrophages and especially MCs to contain large amounts of AA. In fact, 23±6 and 45±9% of the total cellular AA is stored in the neutral lipid fraction of macrophages and MCs respectively, with ~80% of the AA in neutral lipids residing in TAGs in both cell types [43]. However, neither the function(s) nor the mode of utilization of AA from the large TAG pool has been defined as yet.

**Table 1:** MCs in which lipid droplets have been observed and/or studied

Species	Source	Reference(s)
Human	MCs from lung tissue	[36,37,83]
	MCs derived from cord blood CD34^+^ progenitors	[58]
	MCs derived from peripheral blood CD34^+^ progenitors	[55]
	MCs (LAD 2) originally derived from bone-marrow-derived progenitors of an MC sarcoma/leukaemia patient	[55]
Mouse	MCs derived from bone marrow progenitors	[86]
Rat	Rat basophilic leukaemia cell line (RBL-2H3)	[86]

As will be discussed in the next section, the identification of TAG-hydrolysing enzymes in LDs [16,17,28] is compatible with the proposal that AA from TAGs of LDs could be an indirect or direct source for cellular eicosanoid synthesis. This possibility is particularly relevant in the context of the significant amount of eicosanoids produced and secreted by activated MCs [31,44]. These recent findings suggest that the LDs of MCs constitute a new tool for studies of the regulatory mechanisms focusing on AA metabolism, eicosanoid biosynthesis and the release of pro-inflammatory lipid mediators.

## ACTIVATED MCs RELEASE PREFORMED PROTEINACEOUS AND NEWLY GENERATED LIPID MEDIATORS

MCs are highly potent effector cells residing at mucosal, submucosal and perivascular areas throughout the body. The expression of a comprehensive set of different cell-surface receptors and their linkage to multiple signalling pathways participating in the exocytotic pathways enables these cells to rapidly respond to distinct stimuli by acutely releasing highly active mediators, hence regulating inflammation, host defence, tissue remodelling and homoeostasis [45]. Besides their classical role in the development of IgE-mediated acute hypersensitivity reactions, including asthma and other allergic disorders, MCs exert pathophysiological effects in a wide range of different chronic inflammatory diseases, for example atherosclerosis, rheumatoid arthritis and obesity [46]. MCs originate in the bone marrow from pluripotent haemopoietic stem cells, circulate as progenitors and ultimately find their way into the various tissues of the body. Tissue homing of MC progenitors is mainly directed by KITLG [KIT ligand; also termed SCF (stem cell factor)], which is crucial for the development and survival of MCs. Mature tissue MCs are long-lived highly granulated cells whose activation and subsequent exocytosis of granules (degranulation) can be triggered by immunological [for example FcϵRI (high-affinity receptor for IgE) cross-linking] and non-immunological (for example, pathogens and peptides) stimuli, thus leading to granule-mediated modification of the microenvironment of the activated cells [45]. The secretory granules of MCs are filled with various preformed mediator molecules, the best known of which are histamine, serotonin, heparin, the proteases tryptase, chymase and cathepsin G, as well as various cytokines, including IL (interleukin)-8 (reviewed in [45,47]).

**Figure 1:**
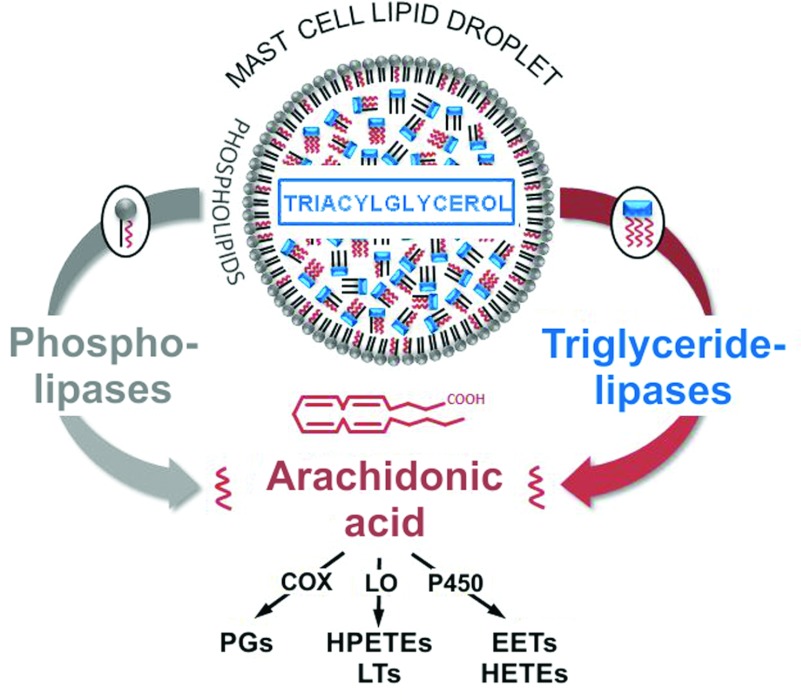
Sources of AA for eicosanoid biosynthesis in human MC LDs MC LDs contain two major sources of AA: PLs as constituents of the LD surface monolayer and TAGs in the core of the LD. Phospholipases and triglyceride lipases mediate the liberation of AA from these two precursor pools to provide substrate for the biosynthesis of eicosanoids (PGs, LTs, HPETEs, EETs and HETEs) via three major pathways mediated by COXs, LOs and cytochrome P450 enzymes (P450). For further details, please refer to the text.

Besides the secretion of preformed mediators during degranulation, MC activation also triggers the release of acutely *de-novo*-synthesized lipid mediators such as eicosanoids, but possibly also growth factors and various cytokines [45]. Compared with the comprehensive knowledge about the identity of preformed proteinaceous mediators in the granules of MCs, our insights into the biology of various lipid mediators are quite limited. In addition, given the lack of knowledge about the mechanisms for their secretion and their unknown, but probably very short, life span, it can be assumed that not all lipid mediators have been identified yet. Among the demonstrated lipid mediators of MCs, eicosanoids play a prominent role. The common precursor molecule for eicosanoids is AA, an ω−6 polyunsaturated fatty acid with 20 C-atoms and four double bonds at positions 5, 8, 11 and 14 (i.e. 5,8,11,14-eicosatetraenoic acid). There are three major pathways of eicosanoid biosynthesis (see Figure 1), in which free AA is oxidized by: (i) COX (cyclo-oxygenase)-1 and/or COX-2 to form PGs, (ii) LOs (lipoxygenases) to generate HPETEs (hydroperoxyeicosatetraenoic acids) and subsequently LTs (leukotrienes), and (iii) cytochrome P450 epoxygenase and ω-hydroxylase to produce EETs (epoxyeicosatrienoic acids) and HETEs (hydroxyeicosatetraenoic acids) respectively [48,49]. It is well established that, upon activation, MCs rapidly generate eicosanoid molecules of three kinds from AA [31]: the prostanoid PGD_2_, and the LTs LTB_4_ and LTC_4_. A recent study reported for the first time comprehensive data about the phenotype-specific production and release of lipid mediators from murine MCs: 90 lipid mediators originating from the three major eicosanoid biosynthetic pathways were characterized by LC (liquid chromatography)–tandem MS [49]. The expression of COX-1 and COX-2 in human MCs is well-studied [48], as well as the expression of 5-LO [50] and 15-LO type 1 [51].

Inasmuch as the eicosanoid mediators can regulate MC functions *in vivo* by autocrine and paracrine mechanisms and the LTs exert pro-inflammatory actions on arterial endothelial cells and smooth muscle cells [52], LT production by arterial MCs may contribute to their pro-inflammatory and pro-atherogenic activities in the arterial wall. This raises the question about the mechanisms by which immunomodulatory cells, including MCs, can promote inflammatory states without inducing a severe allergic reaction or anaphylactic shock due to massive release of inflammatory mediators. Regarding this question, Theoharides et al. [45] have proposed a model for mediator secretion by MCs, which they call ‘differential’ or ‘selective’ release of the mediators, which is not coupled to degranulation. This model of selective release of mediators is also supported by a recent study on mouse BMMCs (bone-marrow-derived MCs) using an *in vitro* model of cell injury in which MCs release LTs, IL-6 and TNF (tumour necrosis factor)-α, but not histamine, due to the binding of fibroblast- and keratinocyte-derived IL-33 to T1/ST2 receptors on the surface of MCs [53].

In contrast, much less is known about the role of LDs as a site of lipid mediator generation. The seminal observation by Dvorak's group [36] in 1983 that LDs (then termed lipid bodies) in lung MCs serve as storage sites for AA was the first to implicate LDs in eicosanoid biosynthesis in MCs. Surprisingly, in the three decades since publication of these results, only fragmentary novel information about the lipid and protein composition of MC LDs has been gained. Interestingly, LDs are found more frequently in MCs residing in regions of inflammation [54]. As the LDs in MCs collectively contain only minute amounts of TAGs compared with adipocytes and hepatocytes, they clearly must function in processes distinct from cellular and/or systemic TAG metabolism. Such processes probably include various intracellular pathways of lipid signalling and the release of lipid mediators. Indeed, the TAGs of LDs may contribute to the pool of AA utilized for MC lipid mediator secretion, as suggested by recent work from our laboratory [55]. This work was facilitated by the establishment of a protocol for the generation of human MCs under well-defined conditions in the absence of serum [56], and is also in accordance with findings demonstrating that human cord-blood-derived MCs contain LDs after *in vitro* differentiation and cultivation in the presence of 10% FBS (fetal bovine serum) [57,58]. Under serum-free culture conditions, we could generate mature and functional human MCs from peripheral-blood-derived CD34^+^ progenitor cells in the presence of KITLG [56]. This protocol does produce exclusively connective tissue-type MCs, i.e. MCs that contain both tryptase and chymase in their granules. Interestingly, differentiation of the isolated CD34^+^ progenitors into mature MCs was found to be accompanied by a steady increase in the number and size of LDs [55]. When we incubated the MCs with unsaturated fatty acids, but not with saturated fatty acids, the formation of LDs was accelerated, and, in agreement with the early findings by Dvorak et al. [36], incubation of MCs with AA led to its efficient incorporation into the TAG pool of the MCs, which we could show to be the predominant lipid class in the LDs of MCs. In fact, by tandem MS, a unique species of TAG molecules containing arachidonoyl residues in all three positions could be demonstrated as components of LDs in MCs [55]. Furthermore, experiments designed to analyse the fate of LDs following stimulus-induced activation of the MCs showed that secretion of cytoplasmic granules was neither associated with a release of LDs nor was the appearance of LDs altered [55]. These results established an axis connecting extracellular AA with its uptake and subsequent delivery to the TAG pool of LDs in human MCs, and provide a basis for future experiments exploring the yet unknown mode of synthesis and release of lipid mediators by inflammatory cells. In the next section, we describe the current status of knowledge about this aspect of the pro-inflammatory function of such cells.

## SOURCES AND SITES OF LIPID MEDIATOR SYNTHESIS IN MCs

Most studies on LDs with human primary cells have been performed with isolated and purified blood leucocytes, notably basophils, neutrophils and eosinophils (reviewed in [35,54,59]). Since the circulating blood does not contain MCs, *in vitro* studies on MC biology require either isolation of MCs from a tissue or isolation of circulating MC progenitors and subsequent induction of their maturation under well-defined culture conditions. The difficulty of either method for obtaining mature human MCs probably explains the paucity of investigations on LDs in human MCs. The early work on LDs in human MCs have been limited to MCs isolated from human lungs, a rich source of MCs [37,43,60–62], but only few observations and/or studies about LDs in MCs derived from other sources have been reported (summarized in Table 1). However, it remains to be seen whether observations made in these cells in fact represent the characteristics of bona fide mature MCs in the respective species. Therefore we will restrict ourselves to studies with mature cells of the myeloblastic lineage (basophils, neutrophils and eosinophils) isolated from blood and/or thoroughly defined MC populations generated *in vitro* from progenitor cells.

Any cell harbouring LDs must maintain an appropriate amount of lipids in its complement of LDs in order to be able to react to extracellular cues in a more or less rapid fashion. As the neutral lipids in the core of the LDs provide fatty acids for a variety of purposes, the fatty acids must be rapidly replenished by re-esterification/re-acylation reactions. The same holds true for PLs in the peripheral layer of LDs. To facilitate cellular lipid homeostasis, fatty acids likely recycle between the PLs and TAGs. The PL molecules derived from the LDs themselves and/or from cellular lipid bilayer membranes and TAG molecules could be resynthesized by acyltransferase-catalysed reactions utilizing PL- and TAG-derived fatty acids respectively. Despite the obvious validity of this metabolic concept, the biophysical problem of how lipolytic enzymes access their substrates within droplets has not been solved. To gain access to the hydrophobic substrates, either the enzyme must translocate its catalytic portion through the PL monolayer or a core lipid domain must emerge on the surface of the monolayer to become accessible to the lipase. It appears that the access of lipases to the LD core is regulated, at least in part, by PAT proteins. For instance, it has been shown that perilipin phosphorylation may be crucial for allowing HSL (hormone-sensitive lipase) to gain access to its substrates [63].

The catabolism of PLs can occur by employing a wide variety of phospholipases. The bulk of literature states that AA is mobilized by the action of various cytosolic and secretory enzymes belonging to the superfamily of the PLA_2_ (phospholipase A_2_) enzymes [64], which have been characterized as hydrolysing the *sn*-2 fatty acyl bond of membrane glycerophospholipids. Depending on the stimulus [65], PC (phosphatidylcholine), PE (phosphatidylethanolamine) [66,67] and PI/PS (phosphatidyl-inositol/phosphatidylserine) [65,68] are important sources for AA production among the PLs, although the localization(s) of these PL pools has not been determined. However, PL species which are not prominent components of membranes, such as PA (phosphatidic acid) [69], MAGs (monoacylglycerols) and even lysophospholipids (reviewed in [70]), have also been proposed as AA donors in IgE-receptor-mediated activation of MCs [71]. The best-studied type of PLA_2_s, cPLA_2α_ (cytosolic PLA_2_; also termed group IV PLA_2_), liberates AA from the ER, phagosomal membrane and the nuclear envelope, but, as claimed in [44], not from the plasma membrane. It is important to note that, although the association of cPLA_2_ with LDs has been amply demonstrated, lipolysis by this enzyme of PL molecules in a surface lipid monolayer, such as that on LDs, has yet to be proven directly. In this context, sPLA_2_s (secretory PLA_2_s) have been reported to be active also intracellularly, i.e. in the course of their secretion [72]. Since these enzymes also act on the PL monolayer of lipoprotein particles, it is possible, by inference, that not only sPLA2s, but also cPLA2s can act on the single PL leaflet of LDs [73] (see Figure 1).

Regarding the relevant AA pathways in MCs, there is uncertainty about the identity of the enzymes involved in production of the substrate AA. Although cPLA_2_ has been identified as an essential player(s) in the production of eicosanoids [69,74,75], the broad spectrum of enzymes with PLA_2_ activity that have been discovered [76] makes it difficult to unambiguously identify any particular PLA_2_ enzyme(s) as a regulator(s) of the MC response involving AA. For instance, one of the three cPLA_2_s in MCs shows a preference for several PL species bearing AA in the *sn*-2 position [77], whereas PI (over PC) was suggested to be the preferred substrate for certain cPLA_2_s [68]. In the case of cPLA_2α_, the PA produced by hydrolysis of PLs by PLD (phospholipase D) appears to be the major, but not exclusive, substrate for the enzyme [69]. Granata et al. [60] have reviewed studies on PLA_2_s, a complex group of enzymes released at low levels from immuno-modulatory cells that include human lung MCs. Interestingly, the sPLA_2_s released from macrophages may also activate MCs and induce their degranulation in a paracrine fashion. Actually, Fonteh et al. [78] were the first to report that immunologically activated murine BMMCs release sPLA_2_ activity that contributes to AA production and eicosanoid biosynthesis. Subsequently, these observations were extended by showing that murine BMMCs express all members of the group II subfamily of sPLA_2_s [79]. However, Fonteh et al. [78] stated that, as in macrophages, it is doubtful that sPLA_2_s can mobilize AA from the plasma membrane of MCs, in agreement with the above-mentioned claim by Bozza et al. [44]. In a paper by Kikawada et al. [80], cPLA_2α_ in combination with sPLA_2_ as an amplifier was shown to regulate TLR2 (Toll-like receptor 2)-dependent eicosanoid production in murine MCs, but, again, the substrate(s) of these enzymes was not elucidated. In another study employing human neutrophils [81], the internalization of sPLA_2_ subsequent to its binding to cell-surface heparan sulfate proteoglycans was reported to be important for the clearance of the cell-surface-bound enzyme, but appeared not to be directly linked to eicosanoid production. Finally, human group V sPLA_2_ was suggested to induce group IVA cPLA_2_-independent cysteinyl-LT synthesis [82].

The evidence reviewed above is based on the dogma that PLs are by far the predominant, if not sole, source of AA for eicosanoid generation. However, this view may need to be modified in the light of several findings. In agreement with the, not yet proven, proposal that LDs contain membrane structures harbouring mostly ER-derived enzymes in their interior, eicosanoid-forming enzymes have been immunolocalized within LDs (reviewed in, for example, [16,44]). Specifically, PGHS (PGH synthase) 1 and PGH2 (also called COX-1 and COX-2) and LOs (5-LO, and in some cases 15-LO) have been localized to LDs in a variety of cells [44], and also in MCs [83]. The presence of these enzymes in LDs would allow for the focal formation of eicosanoids within LDs and the utilization of the substantial amounts of AA in the TAG pool of the LDs [44]. In such a setting, acylglycerol-hydrolysing lipases have been suggested to first liberate AA from TAGs for incorporation into PLs, from which PLA_2_ activities would then release the acid for conversion into eicosanoids. Alternatively, the MC activation process, characterized by a rapid production and release of eicosanoids, would be considerably accelerated by bypassing the intermediate step described above, i.e. when the AA released from TAGs could be immediately used as a substrate for COX/LO enzymes (Figure 1). The most obvious candidate lipases for acting on the acylglycerols for direct release of AAs from TAGs, DAGs and MAGs appear to be ATGL (adipose triglyceride lipase), HSL and MGL (monoglyceride lipase), which are rather ubiquitously expressed enzymes. In fact, ATGL has already been shown beyond doubt to have a crucial role in initiating the lipolytic cascade of TAGs in adipocyte LDs [84]. However, the mode of fatty acid liberation from LDs by ATGL may vary widely among different cell types.

Detailed *in vitro* and *in vivo* studies on the stereoselectivity of ATGL, HSL and DGAT2 (DAG-acyltransferase 2) suggested a ‘three-pool’ model for cellular DAGs in mouse white adipose tissue [85]. Interestingly, ATGL alone specifically generates *sn*-1,3 DAGs, whereas, in the presence of its co-activator CGI-58, it also produces *sn*-2,3 DAGs. Moreover, DGAT2 was identified to associate with LDs, where it prefers ATGL-derived *sn*-1,3 DAGs for re-esterification into TAGs. It is proposed that, at least in adipocytes, ATGL-derived *sn*-1,3 and *sn*-2,3 DAGs are either substrates for HSL-mediated catabolism or for DGAT2-mediated TAG synthesis, but do not serve directly as a substrate for PL biosynthesis or PKC (protein kinase C) activation [85]. To investigate whether ATGL is also involved in LD lipolysis in other human cell types, Smirnova et al. [18] modulated the level of the enzyme in HeLa cells by RNA interference or by overexpression of wild-type and a mutated form of GFP (green fluorescent protein)-labelled ATGL respectively. LD size was inversely correlated with ATGL activity present in the cells, indicating that the enzyme probably is, in addition to adipocytes, also a key enzyme for LD catabolism in other cell types. Interestingly, ATGL is a member of a family of lipases with high homology to serine esterases including the PLA_2_s (Figure 4 in [18]). In particular, an invariant serine residue in the core of the catalytic domain is found in ATGL and human cPLA_2_.

A recent report [86] implies that LD content in murine MCs (the cell line RBL-2H3 and murine BMMCs) is regulated differently from that in adipocytes (3T3-L1). Although the authors [86] suggested that lipogenic stimuli, such as insulin, initiate the formation of LDs in both adipocytes and MCs, the TAG content of MCs, as measured with a TAG quantification kit, did not increase as much as that in adipocytes. This finding is interpreted to indicate that LDs of lipogenesis-induced MCs do accumulate lipids that are different from those in adipocytes. However, lipid staining of insulin-treated cells with Oil Red O revealed massive accumulation of neutral lipids in LDs of MCs. Furthermore, the authors interpreted their findings to support the claims that eicosanoid synthesis is localized to LDs, but such an implication was not supported by direct analysis of the protein content of these organelles [86]. Thus the experiments described above do not exclude the possibility that LDs contribute to eicosanoid production from TAG-derived AA. In fact, studies from our laboratory have shown that mature human MCs obtained from peripheral-blood-derived CD34^+^ progenitors form LDs enriched in AA-containing TAG species following incubation with the precursor fatty acid [55]. Significantly, the secretion of PGD_2_ from these cells within 5 min after IgE/anti-IgE stimulation was attenuated by knocking-down HSL or ATGL alone and most extensively when both enzymes were knocked down (A. Dichlberger, S. Schlager, W. J. Schneider and P. T. Kovanen, unpublished work).

Although the preliminary data described above are compatible, although unlikely, with a direct contribution of TAG-AA for eicosanoid synthesis, the transfer of AA from the LD TAG pool to the LD PL monolayer and subsequent release from the monolayer by PLA_2_ within a few minutes must be studied experimentally. This requires manipulation of the PLA_2_(s) involved, which, unfortunately, have not been unambiguously identified (see above). However, the release of AA from TAG molecules requires the reacylation of the DAG produced, a reaction involving the ATP-consuming activation of the fatty acid to acyl-CoA by long-chain ACSLs (acyl-CoA synthetases) [87]. Interestingly, even though reacylation may not occur in the same time frame as hydrolysis, siRNA (small interfering RNA)-mediated gene silencing of ACSL3 and ACSL4 in MCs also reduces the release of PGD2 (A. Dichlberger, S. Schlager, W. J. Schneider and P. T. Kovanen, unpublished work), which again supports the notion of a direct contribution of the TAG pool of MC LDs to PGD_2_ secretion. These experiments provide the basis for future studies on the enzymology and regulation of PGD_2_ synthesis, and on the identification of the sources of AA used for conversion into eicosanoids by activated MCs.

## HUMAN MCs IN THE PATHOGENESIS OF EICOSANOID-RELATED INFLAMMATORY DISEASES

Regarding the potential clinical significance of the studies discussed in the present review, we wish to emphasize that MCs are among the major producers of eicosanoids, thus contributing to the development of several inflammatory conditions, including asthma, rheumatoid arthritis and atherosclerosis [88]. In particular, MC-derived PGD_2_ has been recognized as an important mediator in asthma by causing bronchoconstriction, vasodilation and alteration of capillary permeability [89]. Recent observations have demonstrated that PGD_2_ regulates T_H_2 (T-helper type 2) inflammation in bronchial asthma via the specific interaction with its two major receptors D prostanoid receptor (DP) and chemoattractant receptor homologous molecule expressed on T_H_2 cells (CRTH2) [89].

Activated MCs are also present in atherosclerotic segments of human coronary arteries [90], and a role for MC-derived eicosanoids has been suggested to contribute to the pathogenesis of human cardiovascular diseases. Both, prostanoids (e.g. PGD_2_ and its metabolites) and LTs (e.g. LTB_4_ and cysLTs) have been reported to promote atherosclerosis [91] and abdominal aortic aneurysm [92]. An aspect of clinical importance is the emerging concept that resolution of inflammation requires a biosynthetically active process utilizing polyunsaturated fatty acids [i.e. AA, EPA (eicosapentaenoic acid) and DHA (docosahexaenoic acid)] to produce specialized pro-resolving mediators [93]. Although these components have not been studied, nor any relevant process investigated in MCs, it appears plausible that such pathways may operate in these cells, providing them with the capacity to contribute to the reinstatement of homoeostasis, as well.

Thus a detailed molecular understanding of the generation of MC-derived eicosanoids may help us to design drugs that block pathophysiologically critical components of the AA pathway in MCs, which may emerge as novel targets in the treatment of chronic inflammatory diseases, such as asthma and atherosclerosis.
